# Wrap it up: myelination of transplanted neurons for repair

**DOI:** 10.3389/fncel.2025.1635551

**Published:** 2025-10-08

**Authors:** María Fernanda Martínez-Reza, Magdalena Götz

**Affiliations:** 1 Division of Physiological Genomics, Biomedical Center, Ludwig-Maximilians-Universität, Munich, Germany; 2 Institute of Stem Cell Research, Helmholtz Center Munich, Munich, Germany; 3 Graduate School of Systemic Neuroscience, Ludwig-Maximilians-Universität, Munich, Germany; 4 SYNERGY, Excellence Cluster of Systems Neurology, Ludwig-Maximilians-Universität, Munich, Germany

**Keywords:** neuronal transplantation, myelination, oligodendrocytes, CNS repair, neuronal graft, neuronal replacement, neurodegeneration, stroke

## Abstract

Degeneration or damage of neuronal circuits in the central nervous system can lead to an irreversible loss of neurons and function in the affected brain region. Neuronal transplantation is a promising therapeutic approach consisting of introducing healthy cells into the damaged or diseased regions to restore lost circuits. To achieve successful neuronal transplantation, proper integration of the graft in the host circuitry is necessary. This includes the restoration of connectivity as well as the recapitulation of the physiological characteristics of the lost endogenous neurons. An often-overlooked aspect to assess the integration of transplanted neurons is the acquisition of cell-extrinsic features, such as myelination. This review explores the interaction between transplanted cells and endogenous oligodendroglia, the evidence of myelination in different neuronal transplantation models, and the checkpoints that can influence graft myelination in the injured or diseased brain. Additionally, it discusses how appropriate myelin ensheathment could help overcome some challenges faced in the field of neuronal replacement.

## 1 Introduction

A range of neurological conditions, including traumatic brain injury (TBI), stroke, and neurodegenerative disorders, result in irreversible loss of neurons and axonal demyelination, both of which contribute to impaired brain function. The idea of replacing these lost cells with exogenous neurons or neuronal progenitors to restore brain function dates back to the early decades of the last century and has been developed through extensive research and clinical trials reviewed elsewhere (Parmar and Falk, 2025; Harary et al., 2023; Yamanaka, 2020; Björklund and Parmar, 2020; Grade and Götz, 2017). The neuronal transplantation field has explored a number of donor cell sources that are directly delivered into the injured or diseased area. These include fetal neurons (Barker et al., 2013), embryonic-derived neural stem cells (eNSCs) (Uchida et al., 2000; Ourednik et al., 2002; Tamaki et al., 2002), embryonic stem cells (ESCs) (Thomson et al., 1998), and more recently, induced pluripotent stem cells (iPSCs)-derived neurons (Takahashi and Yamanaka, 2006; Yamanaka, 2020). While each of these exogenous sources offers distinct advantages (see section 4.2.1), they all encounter the significant challenge of integrating into highly complex adult neuronal networks, which have been meticulously refined during development and are functioning under the straining conditions imposed by neurodegeneration and/or disease at the time of the intervention. The complexity of this process partly explains the variable outcomes that neuronal transplantation studies have encounter in laboratory and clinical trials (Kirkeby et al., 2025). Therefore, in addition to studying the properties and manufacturing of the donor cells, the field is allocating more efforts towards understanding the interactions between the newcomer cells and the host environment: how the transplanted neurons (tNs) can survive and develop within adult circuits devoid of developmental cues, how the different disease and injury environments can shape the tNs integration, and in turn how the environment is reciprocally influenced by the graft. In this review we focus on the interaction between tNs and endogenous glia, with particular attention to the oligodendrocyte lineage cells (OLs). We review the extent to which myelination of tNs is attained, the checkpoints in different host environments that could influence OLs interaction with tNs, and how focusing on the myelination of repaired circuits could improve the outcome of neuronal replacement.

## 2 Myelination and remyelination in axonal regeneration

To attain proper functional and structural integration, tNs must recapitulate both cell-intrinsic and cell-extrinsic aspects of the endogenous circuits, which requires tNs to interact with the surrounding endogenous neurons and glial cells. Analyzing circuit integration using neuronal tracing techniques, including the gold-standard rabies virus (RABV) tracing, has become a key method for understanding interactions between tNs and endogenous neurons (Grade and Götz, 2017). By mapping the input connections to tNs, several studies have revealed how significantly different host environments can influence graft integration and survival (Grealish et al., 2014; Falkner et al., 2016; Adler et al., 2017; Cardoso et al., 2018; Palma-Tortosa et al., 2020; Besusso et al., 2020; Grade et al., 2022; Thomas et al., 2022). Nevertheless, tN-endogenous glia interactions, and in particular oligodendroglia, remain largely understudied and constitute a crucial hurdle to graft integration.

OLs are responsible for myelinating axons in the central nervous system (CNS) and offer crucial metabolic support to their associated axons, a role that evolved prior to the development of myelin in vertebrates and has been conserved throughout evolution (Simons et al., 2024). Myelination establishment involves several highly regulated stages that include the specification of oligodendrocyte precursor cells (OPCs), their migration to target areas, and their terminal differentiation into mature myelin-forming OLs (Bergles and Richardson, 2016). Newly differentiated OLs extend their processes and quickly rearrange their membranes to generate internodes around multiple axons through a series of coordinated steps that include: (1) identification and signaling with the target axons, (2) axonal wrapping with membrane outgrowth, (3) transporting membrane components, (4) compacting myelin sheaths, and (5) node establishment (Simons et al., 2024). After this highly dynamic phase, both OLs and internodes become stabilized and mostly persist throughout their lifespan, albeit undergoing continual renewal of their membrane and structural remodeling (Osso and Hughes, 2024). Our understanding of the axonal cues that determine whether an axon will become myelinated remains limited (Simons and Lyons, 2013). The current model suggests that *de novo* myelination is regulated by a combination of pro-myelinating cues and the removal or absence of inhibitory cues (Redmond et al., 2016).

Both developmental myelination and adult remyelination aim to achieve the same fundamental goal: wrapping axons in myelin sheaths. Remyelination is the process by which myelin sheaths are restored following demyelination. Therefore, it may be hypothesized that these processes could utilize similar mechanisms, i.e., the “recapitulation hypothesis” of remyelination (Franklin and Hinks, 1999). While developmental myelination and remyelination follow parallel steps, including OPC proliferation, migration, and differentiation, the physiological conditions involved are abysmally different. Myelin regeneration faces several specific challenges including a highly inflammatory environment hostile for newly formed OPCs (discussed below) and the less plastic adult/aged OPCs. Nevertheless, in neuronal replacement and axonal regeneration, newly generated axons must signal the host environment to acquire *de novo* myelination in a pathological condition. Whether the mechanisms that play in these exceptional circumstances resemble more closely developmental or adult remyelination is poorly understood.

Studies on axonal regeneration upon injury have shown that myelination of new axons is required to establish functional connections, particularly if the axon was myelinated prior to injury (Bei et al., 2016; Alto et al., 2009; Marin et al., 2016; Wang et al., 2020). Importantly, the myelin ensheathment of new axons is not always induced and varies according to the injury environment and the treatment (Hilton and Bradke, 2017). For instance, Bei et al. (2016) have shown that newly regenerated axons from retinal ganglion cells (RGCs) are able to form adequately targeted synapses, however, as these new axons fail to myelinate, they do not conduct action potentials (APs) and fail to restore visual function. In the follow-up study, Wang et al. (2020) showed that upon optic nerve injury, OPCs fail to differentiate into mature myelinating OLs and the combined manipulation of the OPCs’ GPR17 signaling pathway and microglia led to extensive myelination of the regenerated axons. Similarly, Alto et al. (2009) demonstrated that the expression of neurotrophin-3 facilitates sensory axon regeneration and synapse formation, but the axons remain unmyelinated. In contrast, Marin et al. (2016) described that after Pten knockout, cAMP administration and inflammatory stimulation, regenerating RGC axons are myelinated and assemble nodes of Ranvier. Altogether, the myelin ensheathment of new axons is essential to attain a complete functional recovery and the set of signals that prompt this process remains to be fully elucidated.

## 3 Adult transplantation and evidence of myelination

The functionality of the connections established by neurons transplanted into the adult injured brain has been probed, by methods including electrophysiology (Tornero et al., 2017) and calcium imaging (Falkner et al., 2016). These studies have shown that, under certain injury conditions, tNs can develop appropriate receptive field properties, demonstrating functional characteristics comparable to those of endogenous neurons (Falkner et al., 2016). Moreover, studies using optogenetic and chemogenetic tools combined with behavioral analyses have shown that functional recovery is closely linked to tN activity (Steinbeck et al., 2015; Yu et al., 2019; Zhu et al., 2019; Palma-Tortosa et al., 2020; Andreoli et al., 2020). While these functional outcomes suggest robust integration, a critical gap remains in knowing whether this is achieved by adequately myelinated tN-derived axons. Some studies have described axonal myelination of tNs through immunohistochemistry and electron microscopy (see Table 1), yet these findings often show a single example and lack quantitative analysis, as well as failing to show adequate co-localization when using light microscopy. Key questions, such as when and how these axons become myelinated, remain largely unanswered. In the following sections, we will review the available evidence on tN axon myelination and discuss its implications for functional integration and repair.

**Table 1:** Summary of studies showing examples of myelinated tN axons (organized by region in chronological order).

Region	Type of donor cells	Model transplanted	Analysis timepoint	Type of myelination observed	References
Basal ganglia	Fetal striatal graft (E14-E15 rat embryos)	IBO in caudate-putamen adult rat Transpant: 1–2 wpi	5–16 mpt (rat graft)	Single EM example of PHAL-traced striatopallidal tN axons myelinated	Wictorin et al. (1990)
Basal ganglia	Human fetal mesencephalic dopaminergic neurons	Clinical trial. PD patient transplant into putamen and caudate nucleus	11–16 ypt	One image of Luxol fast-blue staining at low magnification shows some neurons with myelin at the graft area. No individual axons shown. Not clear if host or tN axons are myelinated	Li et al. (2008)
Basal ganglia	Human fetal LGE cells	Clinical trial. HD patient transplant into putamen	10 ypt	One IHC image of MBP staining from the lesion/transplant at low magnification shows focal MBP signal at the graft site	Keene et al. (2009)
Spinal cord	Human fetal-derived CNS stem cells grown as neurospheres (hCNS-SCns)	T9 contusion injury adult NOD-*scid* mice. Transplant: 9 dpi (Cummings) and 30 dpi (Salazar)	1 and 4 mpt	IHC for MBP and Caspr and immuno-EM detection of graft-derived OLs myelinating some host axons. Not clear if graft axons are also myelinated	Cummings et al. (2005) and Salazar et al. (2010)
Spinal cord	hESC-derived motor neurons and OPCs	T8 complete transection adult rat. Transplant: acute	4 mpt	One IHC micrograph shows a couple of tN NF70^+^ fibers surrounded by APC^+^ myelin signal	Erceg et al. (2010)
Spinal cord	MEF-derived iPS-secondary neurospheres (iPS-SNSs)	T10 contusion injury adult wt and *shiverer* mice. Transplant: 9 dpi	2 mpt	One image of Luxol fast-blue staining at low magnification for grafts in wt mice and IHC MBP^+^ EM for grafts in *shiverer* mice. Graft-derived OLs myelinating host axons. Not clear if graft axons are also myelinated	Tsuji et al. (2010)
Spinal cord	Fetal striatal NPCs (E14 mouse embryos)	T10 contusion injury adult mice. Transplant: 9 dpi	6 wpt	IHC for MBP and immuno-EM detection of graft-derived OLs myelinating host axons. Not clear if graft axons are also myelinated	Yasuda et al. (2011)
Spinal cord	Fetal spinal cord NPCs (E14 Fischer 344 rat embryos) embedded into fibrin matrices containing a cocktail of growth factors	T3 complete transection, Fischer 344 adult rats. Transplant: 2 wpt	7 wpt	One example IHC for MAG and immune-EM detection of graft-derived GFP^+^ axons in host white matter myelinated by host OLs	Lu et al. (2012)
Spinal cord^a^	Fetal spinal cord NPCs (E14 rat embryos)	C5 hemisection adult rat. Transplant: 2 wpi	3 mpt	104 GFP^+^ axons examined via EM. 24% myelinated. IHC for APC showed host OL-driven myelination	Hunt et al. (2017)
Cortex^a^	Fetal motor cortex fragment (E14 mouse embryos)	Motor ctx aspiration adult mouse. Transplant: acute	2 mpt	Single IHC micrograph shows double labeling of several GFP^+^ tN axons with PLP in the cortex. Approximately ~30% of GFP^+^ fibers were also PLP^+^ in the cortex and striatum. Variability between samples and how far this picture is from the transplant is not clear	Gaillard et al. (2007)
Cortex	Mouse ESCs-derived cortical cells with visual/occipital cortex identity	IBO in visual or motor cortex adult mice. Transplant: 3 dpi	3–4 mpt	A couple of EM examples of myelinated immunogold-labeled GFP^+^ tN axons. One axon in the cortex and a couple of axons in the thalamus/dLG	Michelsen et al. (2015)
Cortex	Cortically fated human iPSC cell-derived neuroepithelial-like stem cells (If-NES)	dMCAO in adult rats S1. Transplant: 48 hpi	6 mpt	Few EM examples of immunogold-labeled GFP^+^ tN axons myelinated in the ipsi-and contralateral cortex and corpus callosum. Myelin observed at initial, intermediate, and compact myelin stages. No quantification IHC of MBP and human mitochondria shows graft-derived OLs myelinating axons	Palma-Tortosa et al. (2020)
Cortex	Human ES-induced neurons (hES-iNs) overexpressing neurogenin 2 (NGN2)	dMCAO in adult rats S1. Transplant: 48 hpi	3 mpt	Few EM examples of immunogold-labeled STEM121^+^ tN axons myelinated in the ipsi-and contralateral cortex and corpus callosum. No quantification	Martinez-Curiel et al. (2025)

a Quantitative study.

tN, transplanted neurons; E, embryonic day; wpi, weeks post-injury; mpt, months post-transplant; ypt, years post-transplant; IBO, ibotenic acid injection; PHAL, *Phaseolus vulgaris* leucoagglutinin anterograde neuronal tracer; EM, electron microscopy image; PD, Parkinson’s disease; HD, Huntington’s disease; LGE, lateral ganglionic eminence; IHC, immunohistochemistry; MBP, myelin basic protein; C5, fifth cervical vertebra; T10, tenth thoracic vertebra; OL, oligodendrocytes; MEF, mouse embryonic fibroblasts; dMCAO, distal middle cerebral artery occlusion.

While we focus on transplantation in models of adult CNS pathology, it is important to mention that neuronal transplantation has also been used as a tool in experimental biology to probe human neuronal development *in vivo.* These so called “interspecies chimeras” are reviewed elsewhere (Vermaercke et al., 2022) and involve the xenotransplantation of human-derived cells into embryonic/neonatal hosts, which provides an intact, actively developing environment for transplanted cells to integrate. Not surprisingly, studies consistently show that younger hosts support better graft survival and connectivity than older ones (Crutcher, 1990; Thomas et al., 2022; Paterno et al., 2024), and that integration declines progressively with host age, even in the case of homotopic transplantation of rodent donor cells into adult rodent hosts (Greene et al., 2025). However, these findings are difficult to generalize, as graft integration depends strongly on the lesion environment, and near-normal connectivity can still be achieved in the adult brain under certain conditions, such as in the context of synapse loss (Falkner et al., 2016; Thomas et al., 2022). While these studies assessed transplanted cell development and graft-host connectivity as a function of host’s age, the host’s age effects on myelination have not yet been directly quantified. The known decline in OL function with age (Shen et al., 2008; Hill et al., 2018; Neumann et al., 2019) may suggest that older hosts would myelinate grafts less efficiently than younger hosts, but this has not been systematically characterized. Interestingly comparison of human OPCs (hOPCs) transplanted into young adult mice and aged mice showed that both wildtype and modified Neuropilin-1 (NRP1) knock out hOPCs migrated equal distances regardless of the age of the host, suggesting that in this context, the intrinsic properties of the transplanted cells can offset the aging environment (Wagstaff et al., 2024). While older brains seem generally less “receptive” to new inputs, these are also the focus of most studies as there is a greater clinical need for neuronal replacement in adult or elderly patients.

**Figure 1:**
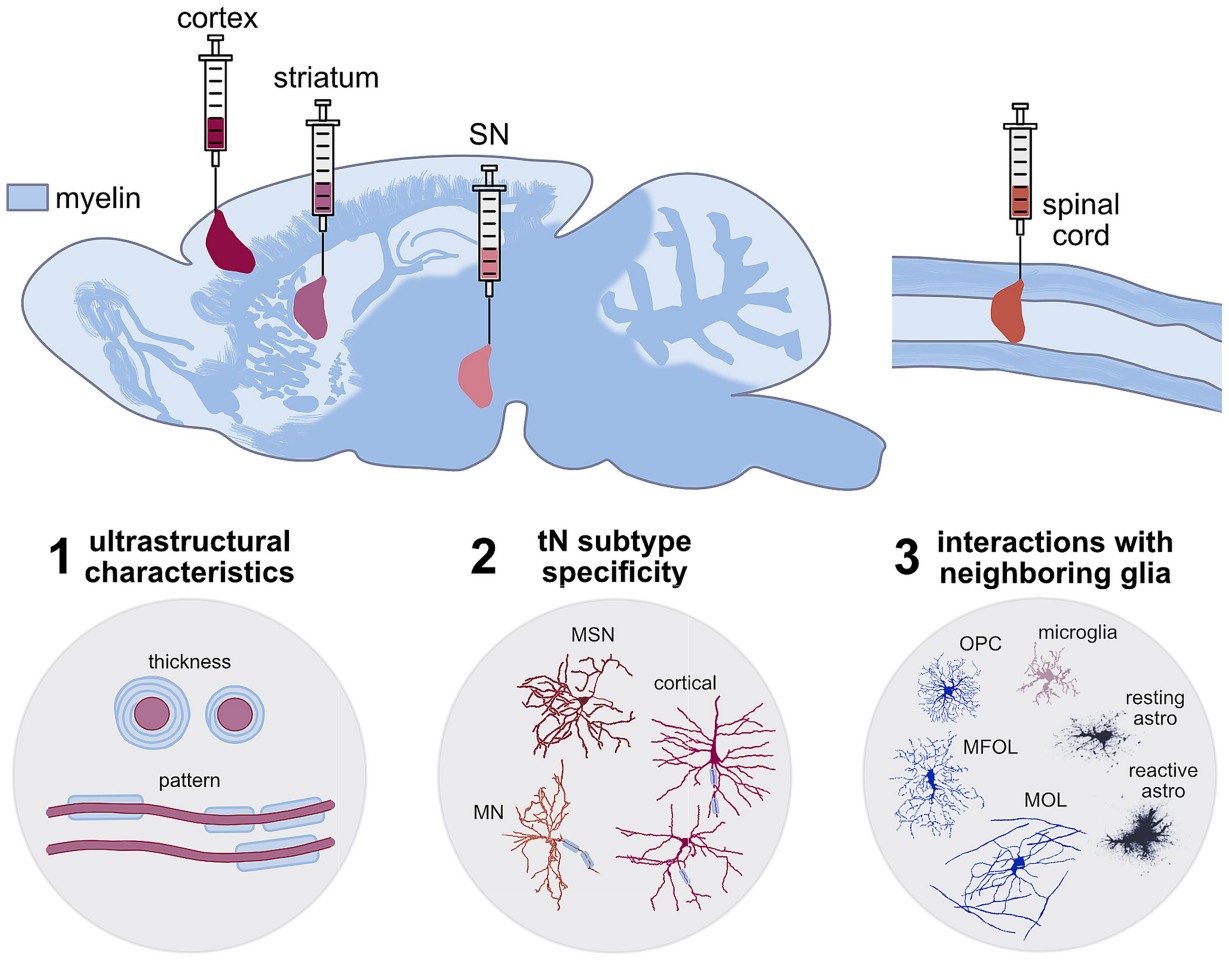
Schematic showing the different complex myelination patterns across the CNS highlighting the regions where neuronal transplantation interventions have been studied after TBI and stroke (cortex—burgundy), SCI (spinal cord—orange), and basal ganglia neurodegeneration (striatum—pink and substantia nigra—pale pink). These tNs have to integrate and acquire myelination at different levels of specificity to recapitulate the host characteristics (1) ultrastructural (myelin thickness and pattern), (2) neuronal subtype-specificity, (3) regional specificity and interactions with other neighbouring host glia. Example neuron and glia traces where adopted from: MSN—medium spiney neurons from striatum and cortical neurons (Oikonomou et al., 2014). Spinal cord motor neuron (MN) from neuromorpho.org (RRID:SCR_002145-NMO_00912) (Li et al., 2005; Tecuatl et al., 2024), Oligodendrocytes: OPC—oligodendrocyte progenitor cells, MFOL—myelin forming oligodendrocytes, MOL—mature oligodendrocytes (Fröhlich et al., 2011), microglia (Fernández-Arjona et al., 2017), resting and reactive astrocytes (Wilhelmsson et al., 2006).

Three of the main CNS targets that have been most explored for neuronal transplantation are the basal ganglia, cerebral cortex, and spinal cord (Figure 1). These regions are commonly affected in various pathologies and injuries that are often spatially confined, such as stroke and spinal cord injury (SCI), or affect specific localized cell types (e.g., Parkinson’s and Huntington’s diseases), making them ideal models for studying local neuronal replacement. The localized nature of the neuronal transplants highlights the importance of considering regional differences across the CNS. Myelination and OLs within the CNS vary both in distribution and composition (Xu et al., 2024; Dimou and Simons, 2017; Foerster et al., 2019) which distinguishes, e.g., white from gray matter, and shows significant heterogeneity across different cortical and subcortical areas (Call and Bergles, 2021). While the cellular and molecular mechanisms governing this regional specificity remain largely unknown, several studies highlight the interaction between Ols and neurons as important drivers of these differences. For instance, ectopically enlarging axon calibers of normally unmyelinated cerebellar granule cells leads to OPC expansion, differentiation and *de novo* myelination (Goebbels et al., 2017). Whether the myelination observed in tNs across these different environments recapitulates the exquisite diversity and specificity of the myelin patterns from the host cortical circuits has not yet been explored.

### 3.1 Basal ganglia transplantation

The basal ganglia comprise a group of subcortical nuclei that form extensive connections with the cerebral cortex, thalamus, and brainstem, and are involved in a wide range of functions including motor regulation, cognitive processing, emotional modulation, and learning (Pott et al., 2009). This group of brain nuclei is of particular interest in neuronal replacement as it hosts the specific cell populations that degenerate in Huntington’s and Parkinson’s diseases. In Huntington’s disease (HD), GABAergic medium spiny projection neurons (MSNs) in the striatum are primarily affected while in Parkinson’s disease (PD) the dopaminergic (DA) neurons in the substantia nigra pars compacta (SNpc) undergo degeneration.

Compared to the cortex and spinal cord, OL function and myelination in the basal ganglia is less studied. However, evidence suggests that this region’s distinctive axonal architecture and neuronal activity patterns have profound influence in OLs both locally and along the axons in the corpus callosum (Caldwell et al., 2023). Notably, axons of MSNs are myelinated (Bishop et al., 1982), while dopaminergic neurons localized in the SNpc are scarcely myelinated despite having long axons (Sulzer and Surmeier, 2013; Orimo et al., 2011; Braak and Del Tredici, 2004). The underlying mechanisms behind this distinction have not been explored, as studies explicitly comparing myelination of SNpc DA neurons with other neuronal types are largely absent. Nevertheless, to understand the contribution of OLs to PD, some reports have focused on studying the interactions of OPCs with DA neurons, even in regions where actual myelin sheaths are scarce. For instance, high-resolution imaging of the mouse midbrain revealed that NG2^+^ OPCs in the SNpc are abundant (in a 1:1 ratio with DA neurons) and their processes frequently envelop both the soma and axons of TH^+^ neurons (Fitzgerald et al., 2025). In addition, it has been shown that midbrain dopamine axons can synapse onto OLs *in vivo* and that some Olig2^+^/PDGFRα^+^ OPCs can express Drd1/Drd2 receptors (Caldwell et al., 2023), implying that dopaminergic fibers can directly signal to OPCs, potentially influencing myelination. Thus, OPCs make extensive axo-glial contacts in SNpc despite minimal myelin.

As SNpc DA neurons are scarcely or thinly myelinated, very few transplantation studies dealing with this neuronal population have assessed whether the grafts acquire myelination or affect the host OL population. Nevertheless, beyond myelination, DA neuron interactions with OLs and OPCs in the SNpc have been suggested to have a prominent role in the etiology of PD. Recent studies have identified a distinct cell type association between PD risk and OL-specific gene expression (Agarwal et al., 2020; Adams et al., 2024). For instance, some of studies have shown that PD-linked risk loci identified by genome-wide association studies (GWAS) associate strongly to OL transcriptional profiles (Bryois et al., 2020; Dehestani et al., 2024). Moreover, a recent single-nuclei multiomic study that profiled gene expression and chromatin accessibility of young, aged and PD human postmortem midbrain samples showed that OLs were both the major cell type in the midbrain and one of the most altered cell types in PD, and identified a “disease-associated” OLs subpopulation (Adams et al., 2024). When comparing differentially expressed genes (DEGs) among healthy and disease-associated OL populations, PD-specific changes included a reduction in multiple genes involved in myelination (i.e., MBP, MOBP, MOG), glial-neuron adhesion (i.e., CTNNA3, NRXN3), as well as an increase in stress-response genes (i.e., HSP90AA1, FKBP5) and well-known neurodegenerative markers, such as MAPT which contains multiple PD-associated single nucleotide polymorphisms (SNPs) (Adams et al., 2024). Furthermore, MRI studies in PD patients have revealed widespread changes in white matter, with most connections emerging from the basal ganglia showing a reduced myelin content (Boshkovski et al., 2022).

The heterogeneous myelin distribution of the basal ganglia and complex OL-DA neuron interactions add an extra layer of complexity to consider in neuronal replacement studies when discussing homotopic versus ectopic graft placement. A direct comparison between intranigral versus intrastriatal grafts using ventral mesencephalon (VM) fetal donor cells (Droguerre et al., 2022) or human ESC-derived midbrain DA neurons (Xiong et al., 2021) after 6-Hydroxydopamine (6-OHDA) injection into the SNpc in adult mice showed that, while both sent projections to appropriate dopaminergic targets, the intranigral grafts promoted better survival of DA neurons and the recovery of fine motor skills, whereas the intrastriatal grafts elicited higher inflammation/gliosis response at the site of the graft (Droguerre et al., 2022; Xiong et al., 2021). While both astrocyte and microglia reactivity were studied and showed an increase in GFAP^+^/C3^+^ and Iba1^+^/CD68^+^ populations in the intrastriatal compared to the intranigral grafts (Droguerre et al., 2022), oligodendrocyte and myelination responses to these transplants were not assessed. Whether or not homotopic or ectopic transplants acquire differential myelination or interact differently with the local OL population has not yet been explored. Encouraging observations have been reported from clinical trials in which fetal mesencephalic tissue was transplanted into the putamen and caudate nucleus of two PD patients. Follow-up assessments conducted after 11–16 years post-transplantation revealed sustained graft survival, with numerous dopaminergic neurons exhibiting long processes and forming dense neural networks within the grafts and the surrounding striatum. Notably, myelinated axons were observed in the grafted regions using Luxol fast blue staining (Li et al., 2008). However, the extent to which this myelination was attained, whether the axons corresponded to DA neurons or other type of tN, and whether it recapitulated the endogenous patterns was not studied.

In addition to the dopamine-based strategies to modify circuit activity in the basal ganglia, the transplantation of cells derived from the medial ganglionic eminence (MGE) has been assessed to alleviate motor symptoms in the 6-OHDA PD model (Martínez-Cerdeño et al., 2010). Following transplantation, MGE cells migrated away from the injection site and spread across the host striatum. By 4 weeks post-transplantation (wpt), ~75% of transplanted MGE cells expressed the mature neuronal marker NeuN and GABAergic markers. Moreover, these tNs exhibited several markers typical of striatal GABAergic interneurons, including as calbindin (CB), calretinin (CR), parvalbumin (PV), and somatostatin (SST). Although striatal interneurons are often myelinated, especially PV^+^ (Lousada et al., 2021), myelination was not specifically assessed in this study. Interestingly, one fourth of the transplanted MGE cells were NeuN negative and CNPase^+^, suggesting that a subpopulation of MGE cells differentiated into OLs (Martínez-Cerdeño et al., 2010). This represents an exciting avenue as MGE grafts could provide both neurons and OLs required to repair the damaged circuit. Whether the transplanted OLs have a preference to myelinate tN axons over host axons has not yet been explored.

Similar to PD, cell replacement therapy for HD has also advanced to clinical trials (Shah et al., 2025). While white matter dysfunctions have been widely reported in HD patients (Back et al., 2024), explicit reports of graft myelination in clinical and pre-clinical studies are scarce. Encouragingly, axonal tracing of fetal striatal transplants grafted into the excitotoxically lesioned striatum of rats revealed that these axons originate predominantly from DARPP-32-positive MSNs and extend exclusively in the caudal direction. The tNs axons form a single, well-defined bundle that follows the trajectory of the myelinated fibers of the internal capsule, ultimately branching into terminals within the globus pallidus. Electron microscopy (EM) analysis identified myelination in a couple of examples of these tNs striatopallidal axons (Wictorin et al., 1990); however, how many tNs have myelinated axons and the extent to which this myelination replicates the endogenous pattern remains uncharacterized. Notably, myelination within the graft site has not been consistently observed and appears to depend on factors such as the type of transplanted cells and the specific HD model studied. For instance, in the Q175 genetic mouse model of HD, human NSCs transplanted into the striatum differentiated into MSN- and interneuron-like cells but did not give rise to observable myelinated tracts under infrared illumination with differential interference contrast (IR-DIC) microscopy. The grafts were readily distinguishable from the surrounding host tissue, which appeared darker due to myelinated fiber tracts. In contrast, the grafts were more translucent and densely populated with diverse cell types (Holley et al., 2023), suggesting limited myelination within the graft. Further characterization is required to confirm this observation. In clinical trials, histological analysis of long-surviving HD graft patients has provided some evidence of possible myelination in graft tissue. In one case report, post-mortem examination 10 years post-transplant showed intense gliosis of bilateral caudate nuclei and putamen, as well as the unfortunate overgrown of masses and a cyst from the transplant. Staining of the graft site and circumscribed masses showed individual and bundled myelinated MBP^+^ axons which rarely appeared to cross the graft-host boundary (Keene et al., 2009). While this report provides evidence that transplant-derived axons can undergo myelination in the human brain, it remains unclear whether in this case this was promoted by the abnormal graft overgrowth observed in the patient. Therefore, these findings should be interpreted with caution.

### 3.2 Spinal cord transplantation

Neuronal transplantation has been extensively studied as a strategy to repair SCI. A key goal is not only to form new relay circuits, but also to achieve proper myelination of the regenerated axons (Roman et al., 2024), since the spinal cord is heavily myelinated. The proportion of myelinated spinal cord axons that has been quantified varies across regions, species, age and techniques used (Saliani et al., 2017) and ranges from 40–60% in rat sacral spinal cord (Chung and Coggeshall, 1983), to an impressive >99% in lumbar mouse spinal cord (Schäffner et al., 2023). In the spinal cord, myelination is more advanced during early postnatal development compared to the brain (Hilscher et al., 2022). While brain and spinal cord OPCs have different spatial and temporal developmental origins, they both emerge from distinct waves in a ventral-to-dorsal temporal sequence (Vallstedt et al., 2005). Interestingly, spinal cord and brain OPCs converge in similar transcriptional profiles, despite their different developmental origins (Marques et al., 2018). On the other hand, mature OLs display notable transcriptional heterogeneity across different brain and spinal cord regions, giving rise to distinct subpopulations that have been characterized via single-cell (sc)-RNA sequencing (Marques et al., 2016; Hilscher et al., 2022; Weng et al., 2025). These transcriptomic subpopulations of mature OLs (MOL1-6) have different spatial distributions in the brain and spinal cord and their abundance fluctuates with age and pathology. For example, in the spinal cord, MOL5/6 population increases with age and shows a spatial preference for gray matter (GM), whereas the MOL2 population is predominantly found in white matter (WM) (Floriddia et al., 2020). This transcriptional heterogeneity is reflected at the functional level both *in vivo* and *in vitro*. For instance, MOLs enriched in the brain subventricular zone express a unique profile of transcription factors linked to development, while spinal cord-enriched MOLs are defined by a specific upregulation of immune-related gene signatures (Weng et al., 2025; Yaqubi et al., 2022). Interestingly, *in vitro* when cultured on synthetic microfibers designed to mimic a myelination substrate, spinal cord OLs formed longer myelin sheaths compared to their cortical counterparts, mirroring internodes *in vivo* (Bechler et al., 2015). Spinal cord OPCs also showed increased migration and differentiation capacities, which are regulated by region-specific factors such as SKAP2, a cytoplasmic adaptor protein involved in cytoskeletal remodeling (Ghelman et al., 2021).

Consequently, spinal cord transplants may be more readily myelinated than basal ganglia or cortical transplants. One of the few transplantation studies that quantitatively evaluated tN myelination showed that fetal rat spinal cord NPC grafts after C5 hemisection displayed myelination by host OLs in around 24% of tN axons emerging from the graft quantified 8–14 mm caudal to the lesion site (C8), with a G-ratio equal to that of axons in the intact spinal cord (Hunt et al., 2017). In addition to the myelination of tNs by host OLs, the contribution of transplanted cells to the pool of OLs and their role aiding remyelination and functional recovery has been shown to be relevant. Human, rat and mouse NSCs, ESCs and iPSCs have been shown to differentiate into both neurons and glia (including OLs and astrocytes) when transplanted into the injured spinal cord (Cummings et al., 2005; Erceg et al., 2010; Tsuji et al., 2010; Yasuda et al., 2011). Transplantation of myelin-deficient shiverer mutant NSCs in SCI showed that both wildtype and mutant NSC transplants prevented atrophic changes in the injured spinal cord and promoted axonal growth, but only wildtype transplants preserved/enhanced myelination and locomotor functional recovery (Yasuda et al., 2011). These data highlight the importance of graft-derived OLs to the transplant outcome. Human fetal-derived stem cells grown as neurospheres (hCNS-SCns) transplanted after SCI can migrate to areas of demyelination and differentiate into graft-derived OLs, which then remyelinate spared mouse axons (Cummings et al., 2005; Salazar et al., 2010). Intriguingly, evidence of human cytoplasmic antigen (SC121^+^) OLs myelinating mouse axons could be found by EM, but SC121^+^ axons from graft-derived neurons were not reported to be myelinated by neither graft nor host OLs (Cummings et al., 2005). In a follow up-study, colocalization of SC121 with the paranodal protein CASPR was observed within the white matter, suggestive of host mouse axons remyelinated by graft-derived OLs rather than myelination of tNs since SC121-positive axons rarely extended into the white matter (Salazar et al., 2010). Similarly, Tsuji et al. (2010) reported that iPSC-derived grafts can differentiate into neurons, astrocytes, and OLs. They observed graft-derived OLs myelinating host axons but did not provide evidence of graft-derived axons being myelinated. When human ESC (hESC)-derived motor neuron progenitors and hESC-derived OPCs are co-transplanted after SCI, myelination of transplanted cells was observed. The human-specific neuronal NF70^+^ fibers were surrounded with APC^+^ hNU^+^ OLs, indicating that transplant-derived OPCs can also myelinate tNS (Erceg et al., 2010). Further research is needed to characterize the individual contribution of graft-derived OLs and neurons to locomotor recovery as well as to understand whether tNs can be equally myelinated by transplant and host OLs in SCI. While tOLs can myelinate host axons, it remains unclear whether they are more efficient at this task than endogenous OPCs or surviving host OLs following transplantation. Importantly, the graft and its surrounding microenvironment differ from the original injury site, in part due to the “bystander effects” exerted by transplanted cells on the host parenchyma (Ben-Hur, 2008; Han et al., 2023; Weber et al., 2025). Donor cells are well known to secrete pro-survival, neurotrophic, and pro-myelinating factors that can enhance the proliferation, survival, and differentiation of host OPCs, thereby improving the overall capacity for endogenous myelination and remyelination (Einstein et al., 2009). However, the opposite has also been observed with the transplant exerting strong and sustained microglia activation on the surrounding host parenchyma (Zarb et al., 2025).

Another critical variable across SCI transplantation studies that can profoundly affect the outcome of the intervention is the extent and position of the injury. Most animal studies have focused on transplantation in sharp, unilateral, incomplete injury models (i.e., hemisection and hemicontusion) which present with limited neuron loss, immune response, and gliotic scarring of injury level (Wilcox et al., 2014). Nonetheless, most clinical trials have prioritized patients with complete SCI (Burns et al., 2003; Bretzner et al., 2011; Wilcox et al., 2014). Importantly, also the extent of myelin pathology differs following contusion or transection SCI (Siegenthaler et al., 2007). It has been observed that contusion injuries produce more widespread structural damage, significant white matter apoptosis, demyelination, limited remyelination, and a strong macrophage response that extends several millimeters beyond the lesion core. In contrast, transection injuries that are more common in transplantation studies, cause a more localized pathology, with apoptosis and demyelination largely restricted to the lesion epicenter (Siegenthaler et al., 2007). Given the variability in host microenvironment across SCI models, it is not surprising that the differentiation outcomes of the transplant also vary widely. Some studies suggest that the harsher the SCI, the more pro-astrocytic the graft niche becomes and less transplanted cells differentiate into neurons or OLs (Cao et al., 2001; Wang et al., 2008; Khazaei et al., 2020; O’Shea et al., 2022). Indeed, after injury Notch ligands (Khazaei et al., 2020) and myelin-associated factors such as Nogo (Wang et al., 2015) are upregulated in the spinal cord microenvironment and bias differentiation of transplanted cells toward more astrocytes. Nevertheless, there are reports after complete transection (Lu et al., 2012) or contusion (Cummings et al., 2005), where an important proportion of the grafts differentiated into mature neurons (20–30%). Thus, the distinct SCI microenvironments, the choice of donor cell sources, and the timing of transplantation are variables that all add to the wide differences observed in the proportions of graft cell populations.

### 3.3 Cortical transplantation

The cortical gray matter exhibits unique myelination patterns that vary widely across different cortical regions, layers (Tomassy et al., 2016; Jokhi et al., 2024; Xu et al., 2024) and neuronal subtypes (Call and Bergles, 2021). This diversity is a hot topic of study in the field of myelination and is reviewed elsewhere (Foerster et al., 2019; Sherafat et al., 2021; Osanai et al., 2022; Weng et al., 2025). In brief, the mechanisms that control cortical myelination heterogeneity are poorly defined, and they are thought to involve a combination of factors. These include distinct signals from specific neuronal subtypes residing in each cortical layer (Jokhi et al., 2024), as well as intrinsic characteristics of OLs, such as their transcriptional and functional diversity (Marques et al., 2016; Floriddia et al., 2020; Pivoňková et al., 2024). Additionally, the developmental and maturation stage of OLs plays a role (Sun et al., 2018). Extrinsic cues from the local microenvironment, including astrocytes, microglia, and the extracellular matrix, also contribute to this complexity (Sherafat et al., 2021). OLs in the cortex are transcriptionally heterogeneous, with distinct subpopulations that preferentially myelinate specific types of axons (Weng et al., 2025; Foerster et al., 2019). It will be very important to understand to which extent this heterogeneity persists with aging, neurodegeneration and after injury. In addition to this intrinsic diversity, neuronal cues play a critical role in initiating and shaping myelination patterns. This is underscored by findings showing that mislocalization of deep-layer projection neurons to upper cortical layers leads to aberrant, ectopic myelination in those regions (Tomassy et al., 2014). Supporting this, recent work by Jokhi et al. (2024) demonstrates that ectopic expression of pro-myelinating signals that are normally associated with heavily myelinated deep-layer neurons, in upper-layer neurons is sufficient to induce myelination in otherwise sparsely myelinated upper layers.

This complexity and heterogeneity in the myelin network of the host circuits may pose a significant challenge to the successful integration of neuronal transplants that has so far been overlooked in the field. Notably, cortical transplants exhibit a high degree of regional integration specificity. Studies have demonstrated that only donor cells with the appropriate cortical regional identity can effectively contribute to the reconstruction of the damaged cortical circuits (Gaillard et al., 2007; Michelsen et al., 2015; Terrigno et al., 2018; Espuny-Camacho et al., 2018). For instance, mouse ESCs-derived tNs with visual cortex identity integrate successfully when transplanted into a lesioned adult visual cortex, however, the same neurons fail to integrate when grafted into the motor cortex, indicating that a match in region identity between grafted and host tissue is crucial for functional integration. EM analysis in this study revealed that some GFP-labeled tN-derived axons were ensheathed by host myelin in both the cortex and dLG following transplantation into the injured visual cortex. However, the extent of myelination was not quantified and only individual examples were shown, leaving the overall degree of myelination uncertain. Although these neurons also survived after transplantation into the motor cortex, their pattern of axonal projections was markedly different, with reduced innervation of the cortex, striatum, and thalamus (Michelsen et al., 2015). Importantly, the study did not report whether the non-specific projections in heterotopic transplants were similarly myelinated. Thus, the extent and specificity of tN myelination remain largely unknown, leaving open critical questions about how selective and region-appropriate myelination can be achieved in diverse transplant scenarios.

Despite extensive preclinical and clinical research in cortical transplantation across various injury models, direct evidence for the myelination of tN axons remains limited. While very few studies have reported the presence of myelinated tN-derived axons following transplantation in the cortex, these observations are often based on isolated examples rather than comprehensive or systematic analyses. This lack of quantification makes it difficult to draw firm conclusions about whether tNs can consistently acquire myelination at all, as well as the specificity, and functional relevance of tN myelination. Moreover, it remains unclear how the myelination of tNs compares to that of endogenous neurons in terms of pattern, thickness and general myelin dynamics. While some studies using models of cortical stroke, aspiration injury, and traumatic brain injury (TBI) have reported myelination of tNs from diverse donor sources, it remains unclear how many of the tNs are myelinated and if these neurons can acquire the complex, region-specific myelination patterns characteristic of the host cortex. One estimation of the extent of myelination that can be acquired by tNs comes from seminal work by Gaillard et al. (2007). In this study, a fragment of embryonic mouse motor cortex was transplanted immediately into an adult mouse brain after a part of the motor cortex was acutely aspirated. At 2 months post-transplant, both EM and IHC analyses revealed myelination of transplant-derived axons in the cortex, striatum, and internal capsule, with approximately 30% of GFP^+^ fibers also colocalizing with PLP. While this study offers valuable insight into the potential for tN myelination, the spatial pattern and variability of myelination across different grafts were not explored, making it difficult to assess the consistency and regional specificity of these findings. Moreover, since analyses were conducted at a single time point, it remains unclear whether the observed myelination is stable over time, or subject to dynamic changes.

Qualitative evidence for myelination of human tNs comes from cortically fated human iPSC-derived neuroepithelial-like stem cells (NES) transplanted into the rat somatosensory cortex following ischemic injury. Upon differentiation these tNs extended axonal projections across both hemispheres, and showed evidence of myelination at early, intermediate, and fully compacted stages, as confirmed by EM. Notably, many MBP^+^ fibers also stained for human-specific markers for mitochondria and STEM121, indicating that graft-derived OLs were functional and actively producing myelin (Palma-Tortosa et al., 2020). However, it remains unclear whether these tOLs preferentially myelinate transplant-derived or host axons, and how many of the tNs were myelinated. These limitations highlight the need for longitudinal, region-specific, and quantitative studies to fully understand the extent, stability, and functional relevance of tN myelination following cortical transplantation.

## 4 Checkpoints in neuronal transplantation myelination/remyelination

The ensheathment of neuronal transplants would require that the machinery for *de novo* myelination is activated and that young/fetal cells are recognized by the adult/aged OL in the host parenchyma amidst the unfavorable environment created by trauma or degeneration. This challenge poses a two-part problem, namely extrinsic OL and intrinsic tN cues must be coordinated. Here we revise some of the checkpoints that can influence the transplant myelination.

### 4.1 Host environment generates extrinsic checkpoints

Numerous studies have shown that the CNS inflammatory response that is upregulated upon injury and neurodegeneration can have detrimental effects on OL differentiation and myelination. OL are highly susceptible to damage under pathological conditions due to several factors (Dent et al., 2015). Notably, OLs operate at one of the highest metabolic rates among brain cell types to support the synthesis and maintenance of extensive myelin sheaths (Meschkat et al., 2022). This leads to the accumulation of harmful byproducts such as reactive oxygen species (ROS) and renders OLs particularly prone to oxidative injury. Further aggravating their susceptibility, OLs exhibit limited antioxidant defenses with low production of glutathione, thereby impairing their ability to counteract oxidative stress (Connor and Menzies, 1996; Thorburne and Juurlink, 1996). Consequently, conditions associated with metabolic or oxidative strain can overwhelm these cells, triggering apoptotic (Dewar et al., 2003) and likely ferroptotic pathways. Additionally, pro-inflammatory cytokines can initiate cell death; interferon gamma (IFNγ) compromises proliferation of OPCs, while tumor necrosis factor alpha (TNFα) induces apoptosis in differentiated OL (Horiuchi et al., 2006; Jurewicz et al., 2005). These cells are also at risk of excitotoxic death resulting from unregulated glutamate and ATP release, which heightens membrane permeability to extracellular calcium and promotes apoptosis (Mcdonald et al., 1998). Such pathophysiological features are hallmarks of conditions that are targets for neuronal transplantation interventions, namely TBI, SCI, stroke, and localized basal ganglia neurodegeneration (HD and PD). Targeting these pathways to improve OL viability might improve transplant integration in these models, however, this might not be a “one size fits all” solution as each of these pathologies present with very different environmental cues, even though commonalities can be found, such as complement activation (Thomas et al., 2022). Moreover, differential inflammatory responses across different brain regions may play a role in regulating localized OL activity. For instance, we have shown that there are profound differences in grey matter glial reactivity, including NG2 glia proliferation, when the white matter is also injured or not (Mattugini et al., 2018).

To better understand the key checkpoints that tNs must overcome to successfully integrate into different brain environments, it is important to consider how integration varies depending on the surrounding pathology. When endogenous neurons are ablated through apoptosis, resulting in minimal reactive gliosis and inflammation, tNs can achieve near-perfect integration with appropriate brain-wide synaptic inputs and functional properties comparable to endogenous neurons (Falkner et al., 2016). However, this level of integration is no longer observed when tNs are transplanted into environments affected by TBI, aging, or amyloid pathology. Using the same brain-wide input connectome analysis, studies have shown that integration is significantly impaired or exuberant under these conditions (Grade et al., 2022; Thomas et al., 2022). These findings highlight the powerful influence of the injury environment on the establishment of synaptic inputs to tNs. For the purposes of this review, we broadly categorize these pathological environments into two groups: acute injuries (TBI, SCI, and stroke) and chronic neurodegenerative conditions (PD and HD). It is important to note, however, that acute injuries often evolve into chronic states, blurring the boundaries between these categories. Finally, it is also important to note that very little to nothing is known about how the transplant affects the progress of the lesion environment.

#### 4.1.1 Acute injury: TBI, SCI and stroke

The pathology following acute injuries progresses through a dynamic continuum of two main phases: a primary injury (such as mechanical trauma in TBI and SCI, or disrupted blood flow in stroke), followed by a secondary injury, which is characterized by a cascade of cellular and molecular events that drive ongoing tissue degeneration. Understanding the distinct processes that define each phase is critical for optimizing the timing and effectiveness of neuronal transplantation, as the host environment’s receptivity to grafted cells may vary across these stages. Moreover, aligning transplantation with phases that favor repair and remyelination may help harness or even redirect endogenous cues to enhance graft integration and therapeutic outcomes.

##### 4.1.1.1 Primary CNS injuries

In the acute phase, the brain microenvironment is characterized by intense inflammation, with the activation of microglia and the release of pro-inflammatory cytokines such as TNFα (Neumann et al., 2002). In addition to the loss of neurons that follow trauma or stroke, acute injuries can lead to demyelination and disrupted remyelination processes (Mahoney et al., 2022). It has been observed that CC1^+^ mature OLs undergo apoptosis in both the acute and chronic phases of TBI (up to 5 weeks post-injury) following a mouse model of cortical-impact injury (Dent et al., 2015). These pathological changes may contribute to secondary axonal injury, and neuronal cell death. Following SCI, spontaneous axonal remyelination is minimal, even though the spinal cord harbors multipotent endogenous spinal neural precursor cells (esNPCs) capable of replenishing lost OLs (Barnabé-Heider et al., 2010). While numerous studies have shown that SCI induces proliferation of esNPCs within the ependymal zone, this proliferative response does not lead to substantial OL differentiation or maturation. Instead, the majority of activated esNPCs differentiate into astrocytes that migrate toward the lesion site and adopt a reactive phenotype, contributing to the formation of a glial scar (Barnabé-Heider et al., 2010). OLs are highly susceptible to damage during the acute phase of ischemia: OL and astrocyte swelling can be observed as early as 30 min post-stroke, with substantial OL death within 3 h (Pantoni et al., 1996). Moreover, it has been shown that just 30 min of oxygen-glucose deprivation can lead to the loss of approximately 90% of OLs within a 9-h period (Tekkök and Goldberg, 2001).

These findings suggest that the microenvironment following acute injury may negatively impact the survival, integration, and myelination of transplanted neuronal precursor or neurons. Interestingly, several studies in which transplants were performed immediately following acute injury, evidence of tN myelination was reported (see Table 1; Gaillard et al., 2007; Erceg et al., 2010). The transplants were performed immediately following acute injury. The robust survival and integration amidst this neurotoxic microenvironment may be explained by the concurrent activation of protective and regenerative mechanisms. For instance, while damage to OLs results in both demyelination and abnormal myelin formation, ischemic events and traumatic injuries also stimulate OPC proliferation and migration (Zhang et al., 2013; von Streitberg et al., 2021). Moreover, growth factors that can support cell survival and angiogenesis are also upregulated at this stage, such as brain-derived neurotrophic factor (BDNF) and vascular endothelial growth factor (VEGF) (Ishizaka et al., 2013; Nakayama et al., 2019). In addition, the upregulation of chemokines in this phase can guide the migration of transplanted cells to the injured area, such as monocyte chemotactic protein-1 (MCP-1) and stromal cell-derived factor-1 (SDF-1) (Lee et al., 2015). Although these findings offer important insights, transplantation immediately after the lesion (Gaillard et al., 2007; Ballout et al., 2016) presents major challenges for clinical translation, as in acute injury scenarios there is often a significant delay between the injury and the availability of transplantable cells. Therefore, the focus of the field is turning towards understanding the interplay between transplants and the subacute/secondary brain injury environment.

##### 4.1.1.2 Secondary CNS injuries

Following traumatic injury and stroke, the wave of secondary cell death, which mainly affects neurons and OLs, spreads rostrally and caudally from the lesion site in SCI, and radially in TBI and stroke, contributing to structural and functional impairments (Carmichael, 2016; Amlerova et al., 2024). Central mechanisms driving this secondary damage include vascular disruption and ischemia, excitotoxicity mediated by excessive glutamate, oxidative stress, lipid peroxidation, and the release of proinflammatory cytokines and signaling molecules, which create a pro-inflammatory microenvironment (Eftekharpour et al., 2008; Amlerova et al., 2024). After injury, spontaneous axonal remyelination is minimal, even in the spinal cord that harbors multipotent endogenous esNPCs capable of replenishing lost OLs (Barnabé-Heider et al., 2010). The subacute phase is marked by a shift from inflammation to tissue repair as the levels of pro-inflammatory cytokines decrease, and anti-inflammatory factors increase, such as interleukin-10 (IL-10). This phase is characterized by the initiation of angiogenesis which can support the integration of transplanted cells into the host tissue. In addition, the expression of growth factors, such as VEGF, can promote the differentiation of transplanted cells into functional neurons and glial cells (Chen et al., 2022). Studies have shown that transplantation in the subacute phase after both stroke and traumatic injury can lead to better functional recovery, as the environment is more supportive of cell survival and integration (Doeppner et al., 2014; Péron et al., 2017; Ballout et al., 2019). Delaying the homotopic transplantation of embryonic cells into the adult mouse motor cortex by one week after aspiration injury significantly improved graft outcomes, including increased vascularization, enhanced cell proliferation and survival, and greater density of tNs axonal projections, and this was accompanied by an increase in the number of astrocytes, microglia, OLs, and CD45^+^ cells, compared to the acute transplantation (Péron et al., 2017; Ballout et al., 2019).

The influence of the state of reactive gliosis onto neural transplants has also been shown by transplantation into GFAP and Vimentin knockout mice which have attenuated reactive gliosis (Pekny et al., 2016). While rodent NSC transplants generally fared better in terms of neurogenesis and proliferation (Kinouchi et al., 2003; Widestrand et al., 2007), this was not the case for human iPSC-derived transplants (Laterza et al., 2018). The different outcomes can be explained on the one hand due to the different host environments, namely intact retinas (Kinouchi et al., 2003) or intact hippocampi on the Rag-1 knock-out background (Widestrand et al., 2007), compared to ischemic cortical stroke combined with immunosuppression by cyclosporine A (Laterza et al., 2018). On the other hand, human and rodent neural progenitors differ in several intrinsic properties including growth requirements (Culbreth et al., 2012), timing of differentiation (Ostenfeld et al., 2002), expression of surface proteins such as aquaporins (Cavazzin et al., 2006), and sensitivity to various compounds (Malik et al., 2014). Moreover, the absence of an effect of reduced gliosis on neuronal differentiation in human NSPCs may reflect species differences in astrocytes. Compared with rodents, human astrocytes are more abundant, diverse, and structurally complex (Oberheim et al., 2009), suggesting that murine reactive astrocytes may interact differently with human versus rodent grafts. Taken together, these findings emphasize that both modulation of host reactive gliosis and donor cell source can shape the outcome of neural transplants, raising intriguing possibilities for how these factors might influence myelination.

Another interesting model to explore with a modified reactive host environment is the depletion of microglia. In response to neural damage and neurodegenerative processes, microglia undergo significant structural and functional changes, enabling them to remove myelin debris and facilitate tissue repair (Safaiyan et al., 2021). In addition, reactive microglia can have a binary influence on OPC dynamics: while microglia activation is required for injury-induced OPC proliferation (Franklin and Ffrench-Constant, 2008; Wang et al., 2020), persistent microglia activation can impair OPC differentiation (Gibson et al., 2019) and delayed ablation of microglia preferentially promotes maturation of early differentiated OPCs into myelinating oligodendrocytes (Wang et al., 2020). Interestingly, microglia depletion markedly enhanced remyelination in a mouse model of chronic demyelination after transplantation of mesenchymal stem cells (MSCs), compared to the MSC transplantation alone (Tahmasebi et al., 2021). Thus, transient microglia depletion after transplantation could be beneficial to promote OPC differentiation and myelination. Nevertheless, microglia ablation can also have deleterious consequences to the transplant integration particularly in the context of proteinopathies. For example, wild-type DA neurons grafted into the striatum of mice overexpressing human *α*-synuclein accumulated more host-derived α-synuclein upon microglia depletion than in control hosts (George et al., 2019). Whether the depletion of microglia has an impact on the myelination of the tNs has not been assessed but the effect is likely to be time and model-dependent.

Taken together, these considerations highlight the importance of precisely timing transplantation to circumvent the highly cytotoxic acute phase post-injury, while concurrently leveraging the endogenous proliferative response of OPCs in the subacute phase. Strategies that combine the delivery of the replacing neuronal cells together with factors that alleviate the inflammation or glial scar and promote OPC differentiation into mature OLs could be beneficial to enhance transplant integration and myelination.

#### 4.1.2 Neurodegeneration: PD and HD models

Historically the basal ganglia circuitry has been a remarkable platform for the development of cell-based repair (Björklund and Parmar, 2020). First, there are a plethora of ablation models available that allow selective damage of the components of the basal ganglia that mimic the pathology seen in humans affected by PD or HD. For instance, the ablation of striatal projections using excitotoxic lesions induced by quinolinic (QA) or ibotenic acid (IBO), and damage to the nigrostriatal dopamine (DA) system using the 6-OHDA neurotoxin. Even before the identification of the HD gene and the development of transgenic models, researchers primarily relied on excitotoxic lesions in the striatum to simulate HD in animal studies (Ramaswamy et al., 2007). Secondly, the striatum contains a well-defined neuronal population (interneurons and MSNs) and strictly unilateral projections, allowing the intact contralateral side to be used as an internal control. Lastly, there exist a battery of tests for striatum-related motor and cognitive behaviors that are perfect for monitoring the behavioral outcomes of the transplantation intervention (Björklund and Parmar, 2020). While the use of these animal models has led to a wealth of discoveries, the translation of this knowledge into clinical applications has yielded inconsistent results (Kirkeby et al., 2025; Parmar and Falk, 2025; Björklund and Parmar, 2020). One possible explanation is that the host environment resulting from a localized excitotoxic lesion differs fundamentally from that of a chronically degenerating brain burdened with aggregated proteins such as alpha-synuclein or huntingtin. In particular, the glial response to these aggregates, as well as the documented uptake of pathological protein aggregates by transplanted healthy cells (Li et al., 2008), underscores the importance of understanding the dynamic interactions between grafted cells and the specific diseased host environment. Our laboratory has demonstrated that transplantation into the amyloid-loaded, intact cortex of a transgenic Alzheimer’s disease mouse model, as well as into the intact aged mouse brain, influences graft integration in host environment-dependent manner. Specifically, both conditions led to increased input connectivity to the graft compared to adult intact controls. Notably, the aged cortex produced a more extensive effect on graft connectivity than the cortex of 8-month-old APP/PS1 transgenic mice, despite exhibiting greater inter-individual variability (Thomas et al., 2022). These findings suggest that while graft survival and integration occur across all tested conditions, the degree of integration critically depends on the specific pathology encountered in the host/patient brain. Therefore, caution is warranted when extrapolating conclusions from one model to another.

The brain microenvironments of HD and PD are characterized by chronic neuroinflammation, posing a persistent challenge for graft survival and function. Post-mortem analysis of HD and PD brains reveal widespread activation of astrocytes and microglia, which secrete pro-inflammatory cytokines and chemokines, fostering a sustained inflammatory milieu and contributing to blood-brain-barrier disruption (Palpagama et al., 2019; Kam et al., 2020). Notably, glial reactivity correlates positively with disease severity. PET imaging confirms microglial activation even in pre-symptomatic HD mutation carriers, increasing with symptom onset (Tai et al., 2007). In HD, astrocytes accumulate mutant huntingtin protein and downregulate glutamate transporters (GLT-1), impairing glutamate uptake and exacerbating excitotoxicity (Khakh et al., 2017). Microglia in the SNpc are particularly prone to a pro-inflammatory profile, potentially contributing to selective vulnerability of dopaminergic neurons (Gosselin et al., 2017). Studies directly comparing transplant integration between excitotoxic lesion models and neurodegenerative transgenic mouse models have revealed striking differences in transplant survival and functional recovery. For example, Zimmermann et al. (2016) compared transplantation of mouse ESC-derived BDNF-expressing NPCs into both a QA-lesioned model and two widely used HD transgenic mouse lines, N171-82Q and the more severe R6/2 strain (Ramaswamy et al., 2007). Interestingly, transplant survival rates were significantly higher in the QA model (20.1%) compared to the transgenic models with survival rates of approximately 17.89% in N171-82Q and only 9.61% in R6/2 mice. These differences correlated with behavioral outcomes: robust motor function improvements were observed in QA-transplanted mice, while only modest effects were seen in both transgenic lines, which highlights the relevance of the host environment in transplants outcome.

Given the challenges posed by chronically neurotoxic host environments, current transplantation strategies are increasingly focused on combining neuronal replacement with the localized or systemic delivery of anti-inflammatory or neuroprotective agents. These combinatorial approaches aim to mitigate the hostile microenvironment, improve graft survival, and ultimately improve functional recovery. Such strategies include engineering donor cells to overexpress neurotrophic factors, such as nerve growth factor (NGF) or brain-derived neurotrophic factor (BDNF) (Martínez-Serrano and Björklund, 1996; Zimmermann et al., 2016; Pollock et al., 2016; Kim et al., 2020) or the continuous administration of neuroprotective/immunomodulatory factors like cerebral dopamine neurotrophic factor (CDNF) either systematically or intracerebrally (Tseng et al., 2022). Particularly in the context of PD, the concern for a limited capacity for long-distance axonal growth in the adult brain, has prompted the investigation of delivering growth-promoting cues in the striatum or the nigrostriatal bundle to guide axonal projections from homotopic transplants placed in the SNpc toward the striatum and reconstruct the lost midbrain DA pathways. For instance, the viral delivery of glial cell line-derived neurotrophic factor (GDNF) to the striatum in conjunction with homotopic transplantation of human iPSC-derived midbrain DA neurons, improved brain-wide mDA target innervation and functional recovery compared to graft-only controls. Notably, caudal and ventral host targets that were poorly innervated by ectopic grafts received more extensive projections under this combined treatment (Moriarty et al., 2022). Another notable example of the modification of the host extracellular environment to improve transplant integration involves the enzymatic degradation (Takeuchi et al., 2013) or the infusion of neutralizing antibodies (Seiler et al., 2017) against inhibitory molecules to facilitate axonal regeneration. The use of high-purity chondroitinase ABC (ChABC), an enzyme that cleaves chondroitin sulfate side chains and effectively degrades chondroitin sulfate proteoglycans known to inhibit axonal growth, has shown encouraging results (Takeuchi et al., 2013). Simultaneous homotopic transplantation of fetal dopaminergic progenitors into the ventral midbrain of 6-OHDA-lesioned mice together with ChABC injected into the nigrostriatal bundle did not affect graft survival, but significantly enhanced axonal growth along the nigrostriatal pathway and increased reinnervation of the striatum compared to untreated grafted controls (Kauhausen et al., 2015).

While several of these studies have examined the glial reactivity around the graft in response to the co-treatments, detailed analyses of the oligodendroglia population reaction and the extent at which these interventions could support the myelination and integration of tN axons, are lacking. Very rarely these studies report tN myelination, although this constitutes a relevant tN integration milestone. Intriguingly, some of the compounds that showed enhanced graft-derived DA fiber outgrowth, for example the Nogo-Receptor 1 antagonist NEP1-40 (Seiler et al., 2017), could also have a positive impact in re-myelination processes (Chong et al., 2012; Petratos et al., 2020). Future studies should focus on evaluating the efficacy of combining cell transplantation with pro-myelinating factors and systematically assess their contribution to axonal integration and functional connectivity. In summary, the host microenvironment plays a decisive role in graft integration. Understanding these disease-specific landscapes and how they diverge from lesion models is essential to tailor cell therapy approaches. Mitigating chronic gliosis while leveraging beneficial glial support will likely improve the functional integration of tNs.

### 4.2 tNs intrinsic checkpoints

While this review has primarily focused on the influence of the host environment on graft survival and integration, substantial evidence also highlights the critical role of the intrinsic properties of transplanted cells. For successful integration, donor cells-whether developmentally immature fetal progenitors or more advanced *in vitro* patterned and matured cells-must adapt to the complex and highly organized architecture of the host neural network. Numerous studies have directly compared various sources of donor cells, such as iPSCs, ESCs and fetal-derived neural progenitors, within the same host models. Such comparative analyses provide valuable insights into how donor cell origin and intrinsic characteristics impact graft viability, differentiation potential, and therapeutic efficacy across different neurological conditions.

#### 4.2.1 Donor cell sources

Fetal derived cells possess a high intrinsic regional identity (e.g., of the brain region they originate from) and a strong innate capacity for target-specific axonal projections making them ideal for accurate neuronal replacement (Grade and Götz, 2017). However, the clinical use of fetal tissue for transplantation poses significant ethical and logistical challenges. These cells are not only difficult to acquire in sufficient numbers but also tend to be heterogeneous, making it challenging to implement standardized, scalable quality control measures suitable for clinical application (Björklund and Parmar, 2020). The isolation of human ESCs in 1998 (Thomson et al., 1998), followed by the development of iPSCs in 2006 (Takahashi and Yamanaka, 2006) marked a major turning point in the neuronal replacement field. Both ESCs and iPSCs allow scalable homogeneous expansion and differentiation under defined protocols. Neurons derived in these cultures can be regionally patterned (Tao and Zhang, 2016) and their integration has been well demonstrated in various pre-clinical disease models. In particular, iPSCs offer the exciting possibility of autologous transplantation of patient-specific cells, thus carrying little risk of graft rejection (Parmar and Falk, 2025).

A direct comparison between hESC and fetal VM transplants in a rat model of 6-OHDA striatum-lesioned rats showed notable morphological similarities between the two graft types at 6-months post-transplantation (Grealish et al., 2014). In both hESC-derived and fetal intrastriatal grafts, TH^+^/GIRK2^+^ neurons were the most abundant subtype. While the proportion of TH^+^, GIRK2^+^ and Calbindin^+^ cells was comparable between hESC and fetal-derived grafts, the total cell numbers were higher in hESC grafts. This total cell count was also higher in intrastriatal grafts compared to homotopic grafts placed in the SNpc, highlighting the importance of the interplay between host environment and donor cells on transplant survival. Notably, human NCAM^+^ (hNCAM) glial cells were detected in grafts from 8-week-old fetal donors but absent in grafts from younger 5.5-week-old fetal tissue and from hESC-derived grafts. Nevertheless, hNCAM^+^ axons were found in all conditions, indicating that glial contribution is not essential for axonal extension. The myelination status of these graft-derived fibers was not evaluated; however, the authors noted the tN axonal trajectories followed both myelinated and unmyelinated tracts. Interestingly, when it came to the long-distance projections from homotopic SNpc grafts, the axonal outgrowth patterns of fetal- vs. hESC-derived grafts differed. Fetal VM tissue was more efficient at innervating the caudate-putamen (A9 DA target), whereas hESC grafts innervated better the infralimbic cortex and septum (A10 DA targets). This target specificity appears to be intrinsically programmed and could be tuned by transcriptional modifications. Grafts derived from a transgenic hESC line overexpressing the transcription factor Otx2, associated with the A10 DA phenotype, showed reduced innervation of A9 targets, emphasizing the critical role of intrinsic transcriptional identity in directing graft integration and connectivity (Grealish et al., 2014).

In contrast, iPSC-derived grafts appear to exhibit greater variability in performance, likely due to the intrinsic heterogeneity of the different donor cell lines. A recent pre-clinical/clinical study assessed the quality of iPSC-derived DA cells from four different patients transplanted into the striatum of 6-OHDA-lesioned athymic rats. Surprisingly, the grafts showed substantia differences in volume, cell density and the total number of TH^+^ cells. Moreover, grafts derived from one patient, failed to show behavioral improvement in rotation tests. This lack of functional recovery was associated with a reduced number of TH^+^ cells and lower striatal TH^+^ fiber density, despite the comparatively larger graft volume (Jeon et al., 2025). These unexpected findings highlight the critical importance of *in vivo* testing each iPSC-derived line and thoroughly analyzing the graft composition, as the complex interactions between graft and host environment cannot be fully recapitulated *in vitro.* It remains unclear whether different iPSC lines differ in their capacity to engage with the host environment, particularly in terms of glial reactivity or recruitment of myelination, and what the donor-specific factors that shape the differential graft outcomes are.

Nevertheless, iPSC-derived cells hold significant promise for neuronal replacement therapies and, in some contexts, may even outperform other donor cell sources. A direct comparison of three human stem cells sources, namely bone marrow-derived mesenchymal stem cells (BM-MSCs), neural progenitors derived from immortalized spinal fetal cell line (SPC-01), and iPSC-derived neural progenitors (iPSC-NPs), transplanted in a rat model of SCI revealed the most significant behavioral improvement in iPSC-NP treated animals. This enhanced recovery was associated with a higher tendency of the iPSC-NP transplant to differentiate into neurons. In contrast, MSC grafts failed to survive 2 months post-transplant, and SPC-01 grafts primarily differentiated into astrocytes, showing only limited graft infiltration of host neurofilaments. Interestingly, the expression of pro-neural factors such as ciliary neurotrophic factor (CNTF) was significantly lower in the iPSC-NP transplanted group compared to the MSC-treated animals. This may help explain the modest locomotor recovery observed in MSC-treated animals despite the limited graft survival (Ruzicka et al., 2017). Similarly, a comparative study of mouse iPSC and ESC-derived neuronal transplants in a rat model of middle cerebral artery occlusion (MCAO) showed a slightly higher number of NeuN^+^ neurons and GFAP^+^ astrocytes in iPSC-treated animals compared to ESC grafts. Functional imaging using positron emission tomography (PET) with ^18^F-fluorodeoxyglucose (^18^F-FDG) showed that both iPSCs and ESCs grafts increased glucose metabolism after ischemia, albeit with different temporal dynamics. In the ESC-treated group, the lesion-to-normal (L/N) uptake ratio peaked at 1-week post-transplantation and was significantly higher than the iPSC and non-grafted groups. However, this ratio gradually declined in the ESC-treated and fell below that of the iPSC-treated group at 4 wpt (Wang et al., 2013). These findings suggest that iPSC-derived grafts may outperform ESCs-derived grafts in certain contexts, however, outcomes are likely to be model- and species-dependent, underscoring the need for case-by-case evaluation.

#### 4.2.2 Donor cell species

While human and mouse OLs show significant overlap in lineage progression, transcriptional regulation and expression of surface markers through lineage stages (Buchet and Baron-Van Evercooren, 2009; Sim et al., 2009; Dietz et al., 2016), myelin development diverge significantly in both timing (Semple et al., 2013) and molecular composition (Gargareta et al., 2022). In humans, gliogenesis begins prenatally and myelination spans years to decades; conversely, in rodents, OL development is largely postnatal and myelination typically concludes within weeks (Craig et al., 2003). On the molecular level, although core myelin proteins such as PLP, MBP, and CNP are conserved, detailed proteomic analyses reveal marked differences: for instance, proteins like PMP2 are specific to human CNS myelin, whereas tetraspanin-2 (TSPAN2) and connexin-29 (CX29/ GJC3) were restricted to mouse myelin (Gargareta et al., 2022). Altogether, these differences reflect underlying disparities in transcriptomic profiles, lineage dynamics and differentiation cues between species that can affect the integration and myelination outcome of xenotransplants.

It has been well established that when human OPCs (hOPCs) or glial progenitor cells (hGPCs) are transplanted into mouse models, they demonstrate remarkable potential to restore myelin with some species-specific kinetics. That is, the intrinsic developmental pacing of human-derived cells remains slower than that of endogenous mouse counterparts, reflecting species-specific regulatory programs that influence migration, differentiation, and myelin maturation (Windrem et al., 2020). In the case of human derived neurons this can become an obstacle as in some instances human-derived tNs have been observed to be unable to recruit murine OLs to myelinate even in dense white matter tracks (Lu et al., 2014; Dell’Anno et al., 2018). While this may be attributable to a lack of inter-species recognition, there are reports showing examples of tN myelination in different injury models such as stroke (Palma-Tortosa et al., 2020; Martinez-Curiel et al., 2025) and different models of SCI (Cummings et al., 2005; Salazar et al., 2010; Erceg et al., 2010) (see Table 1). Therefore, the complex interplay between the injury microenvironment and the intrinsic properties of the donor cells affects the tN myelination outcome, highlighting the importance of analyzing and reporting this feature in future transplantation studies to have a better understanding of the instances that are permissive and beneficial to tNs myelination. Intriguingly, whether tN myelination efficiency or pattern recapitulates species-specific characteristics in the host has not been explored. Moreover, whether tOLs and host-OLs differentially myelinate tNs of different origins and show species-specific affinities, are still open questions. Taken together, these findings emphasize that while rodent models are indispensable for preclinical exploration, species-specific differences in myelination speed, protein composition, and cell regulation must be accounted for when translating results toward human therapies.

#### 4.2.3 Donor cell biological sex

An often-overlooked cell intrinsic aspect that can affect the graft outcome is the biological sex of the transplanted cells. Grafts derived from male fetal spinal cord donors transplanted into female hosts following SCI featured extensive hypervascularization, increased vascular diameter, higher perivascular cell density, and greater T-cell infiltration compared to sex-matched or mixed grafts (Pitonak et al., 2022). This highlights the importance of detailed characterization and reporting of the donor cell biological sex. Whether or not the sex match also influences tN myelination remains to be explored.

#### 4.2.4 Donor cell developmental stage

Although fetal cells remain the most established donor source in the field, factors such as their topographic origin, developmental stage, and biological sex, must be carefully considered, as each can significantly influence how the transplanted neurons integrate into the host environment. For example, the developmental stage at which embryonic rodent NPCs are harvested can profoundly influence graft composition, axonal outgrowth, host axon regeneration, and behavioral outcomes. Seminal work by Gaillard et al. (2003) demonstrated that parietal or occipital E12 cortical progenitors retained a remarkable degree of plasticity when transplanted into lesions where part of the parietal cortex was aspirated. Unlike cells harvested a later stages (E13–E16), which are more developmentally committed (Pinaudeau et al., 2000), E12 donor cells recapitulated the projection pattern of the surrounding neurons following heterotopic transplantation. Similarly, direct comparison of NPCs derived from embryonic days E11.5, E12.5 (dorsal and ventral), and E13.5, revealed significant differences in graft integration after transplantation in SCI (Aceves et al., 2023). Earlier-stage grafts (e.g., E11.5) exhibited greater axon outgrowth, were enriched for ventral spinal cord interneurons and Group-Z interneurons, and enhanced regeneration of host serotonergic (5-HT^+^) axons. In contrast, later-stage grafts (e.g., E13.5) were enriched in late-born dorsal horn interneuron subtypes and Group-N interneurons, supported more extensive ingrowth of host CGRP^+^ sensory axons, and were associated with increased thermal hypersensitivity. Notably, ventral E12.5 grafts exhibited the highest density of Olig2^+^ cells, while other groups showed no significant differences in Olig2^+^ cell abundance (Aceves et al., 2023). Myelination was not evaluated in this study; therefore, it remains to be determined whether higher numbers of Olig2^+^ cells or neuronal densities correlate with differential graft myelination across the different donor cell developmental stages. Whether the developmental stage of the donor cells also influences the host glial reaction or affects the propensity of the grafts to undergo myelination reminds to be determined.

In the case of human-derived iPSCs or NPCs, the differentiation stage of the starting donor cells also plays a role in graft diversification and survival. Generally, a “more defined” starting donor population, such as hNPC-derived neurons, undergoes less proliferation and less astrocytic differentiation compared to undifferentiated hNPCs after transplantation *in vivo* (Fortin et al., 2016). Furthermore, single-cell multiomic and barcode-based lineage tracing analyses have shown that even transcriptomically and epigenomically homogeneous starting hESC-derived DA progenitors can give rise to heterogeneous grafts after terminal differentiation *in vivo* in PD models (Tiklová et al., 2020; Storm et al., 2024). This suggests that longer cultures, sorting and transplantation of postmitotic cells could reduce the variability of the identity of the final graft *in vivo.* However, the sorting process and transplantation of fully mature cells tend to reduce graft survival and outgrowth, resulting in less effective integration (Fricker-Gates et al., 2002; de Luzy et al., 2019; Storm et al., 2024). How the differentiation stage at the moment of transplantation and the final extent of graft heterogeneity *in vivo* may affect the myelination of tN in different host microenvironments has not been explored.

#### 4.2.5 Donor cell activity

Similar to developmental maturation, activity-induced maturation can profoundly affect transplant integration. Several studies have used behavioral (Döbrössy and Dunnett, 2005), optogenetic (Daadi et al., 2016), chemogenetic (Kawai et al., 2021) or a combination (Yu et al., 2019) of these approaches to increase transplant activity. These interventions have consistently boosted survival and synaptic incorporation of grafts across different pathological contexts. Stimulated grafts upregulated genes involved in synaptic plasticity, neuronal differentiation, axonal guidance and downregulated genes for degeneration and inflammation, ultimately enhancing the functional outcome (Daadi et al., 2016; Kawai et al., 2021). Since neuronal activity is a well-studied driver for axonal myelination and myelin plasticity (Gibson et al., 2014; de Faria et al., 2019; Maas and Angulo, 2021), these interventions represent a potent driver of transplant interaction with the host OLs. Notably, Yu et al. (2019) reported that the daily activation of the luminopsin iPS-derived neural progenitors (LMO3-iPS-NPCs) graft that can be noninvasively activated by a luciferase substrate (CTZ) or light, markedly enhanced axonal outgrowth and MBP expression in the peri-infarct stroke region. The authors did not assess specifically the myelination of the tN axons, thus it is not clear whether this is a generalized effect around the transplant site or a specific boost of MBP expression in the activated transplanted cells.

## 5 Discussion: How can myelin be beneficial for neuronal transplantation?

Attaining myelination of neuronal transplants is attracting more attention as this checkpoint could potentially aid not only the recovery of function but also help improving tNs survival, which can still be optimized in pre-clinical and clinical settings. Mounting evidence shows that OLs support neuronal functions and axonal homeostasis in various ways: by providing specialized extracellular vesicles (Chamberlain et al., 2021; Frühbeis et al., 2020) and energy substrates for axonal mitochondria (Lee et al., 2012; Looser et al., 2024), counteracting reactive oxygen species (Kassmann et al., 2007; Mukherjee et al., 2020), and maintaining ion homeostasis (Kapell et al., 2023; Marshall-Phelps et al., 2020). Indeed, OL functions are required to prevent the degeneration of myelinated axons. Moreover, existing host myelin provides a growing scaffold for tNs axons. Both rat E14–derived NPCs and human-derived iPSCs grafted after SCI grew a significantly higher amount of tN axons in the host white matter compared to gray matter. Interestingly, IHC as well as EM analysis showed that 65% GFP-labeled graft-derived axons contacted a myelin membrane per field examined, which was significantly greater than predicted if axons randomly extended through the same fields (Poplawski et al., 2018). While this is promising, much more needs to be done to follow axonal pathways determining if axons are indeed fully or only partially myelinated, if the myelin is compact myelin, and if the appropriate internode length for the respective neuron is achieved.

As the importance of myelination for neuronal physiology cannot be overstated, it is surprising that the neuronal replacement field has developed with little attention to this important aspect. This can stem from different technical and conceptual factors, as a thorough characterization of myelination in tNs requires comprehensive and multifaceted analyses. Firstly, the timeline of *in vivo* studies that envision to assess mature characteristics of tNs would inevitably be prolonged to a few months, particularly in the case of human donor cells (Espuny-Camacho et al., 2013; Martinez-Curiel et al., 2025), which in some cases is not feasible due to constraints on animal experimentation protocols. Secondly, demonstrating that myelin is unequivocally ensheathing tN axons requires the use of high precision confocal microscopy, axonal tracing and find co-localization with more than one immunomarkers for myelin internodes. In addition, the use of complementary ultrastructural analysis with electron microscopy would be necessary, especially in densely myelinated regions. However, to begin addressing whether tNs can achieve *de novo* myelination across diverse environments, injury models, species, and donor cell sources, we advocate for future transplantation studies to include at least one assay assessing myelin co-localization with tNs. Reporting the presence or absence of myelin in these contexts will contribute not only to overcome this major limitation if myelination is not achieved sufficiently, but also to foster better understanding of how myelination of tNs is regulated under varying conditions.
